# CRABEL Score Assessment for Oral Surgery Excision Biopsy Case Notes of Oral Squamous Cell Carcinoma

**DOI:** 10.7759/cureus.57394

**Published:** 2024-04-01

**Authors:** Samyuktha Aarthi, Karthikeyan Ramalingam, Pratibha Ramani, Murugesan Krishnan

**Affiliations:** 1 Oral Pathology and Microbiology, Saveetha Dental College and Hospitals, Saveetha Institute of Medical and Technical Sciences, Saveetha University, Chennai, IND; 2 Oral and Maxillofacial Surgery, Saveetha Dental College and Hospitals, Saveetha Institute of Medical and Technical Sciences, Saveetha University, Chennai, IND

**Keywords:** quality improvement research, quality indicator, administration, quality, clinical audit, discharge summary, consent, initial clerking, crabel scores, oral surgery case notes

## Abstract

Background

Oral surgical records contain all the information regarding a patient, including their history, clinical findings, diagnostic test results, pre-and postoperative care, progress, and medication. Notes that are properly drafted will help the physician argue that the course of therapy is appropriate. Several tools have been created for auditing clinical records; one such tool that may be used for any inpatient specialty is the CRABEL score system developed by CRAwford-BEresford-Lafferty.

Aims

This research aimed to evaluate the oral surgical records using the CRABEL scoring system for quality assessment.

Materials and methods

The case audit was performed from June 2023 to February 2024 for all Excisional biopsy cases of Oral Squamous Cell Carcinoma. Relevant data was retrieved from the Dental Information Archival Software (DIAS) of Saveetha Dental College and Hospitals, Chennai. It was evaluated by two independent oral pathologists trained in CRABEL scores. Two consecutive case records were evaluated. Fifty points were given for each case record. Scoring was given according to initial clerking (10 points), subsequent entries (30 points), consent (5 points), and discharge summary (5 points). The total score was calculated by subtracting the total deduction from 100 to give the final score. The mean scores of the case records were calculated. A descriptive statistical analysis was done with Statistical Package for Social Sciences (SPSS version 23.0; IBM Inc., Armonk, New York). Inter-observer agreement and reliability assessment were made using Kappa statistics.

Results

From the DIAS in that period, the data of 52 cases were retrieved and reviewed. There was no proof of a reference source in the audited records, and one deduction was made to the reference score in the initial clerking, and the effective score was 98 out of 100. The mean values of 52 case records were also 98 out of 100. The observed kappa score was 1.0. There was no inter-observer bias in the scoring criteria. Both observers also gave the same scoring.

Conclusion

Our study illustrates that oral surgery case records in our institution were found to be accurate, as they maintained 98% of the CRABEL score value. Frequent audit cycles will help in standardizing and maintaining the quality of oral surgery case records.

## Introduction

Documentation from oral surgical records serves as the foundation for interprofessional collaboration. As an ongoing, contemporaneous record, it offers details on the care provided, the intended course of treatment, and the outcomes of that provided care. Thorough, clear, factual, and unambiguous documentation creates a reliable, permanent record of the patient and acts as an accurate chronicle of the patient's medical history [1, 2]. Accurate documentation is essential in oral surgery case notes for strengthening the quality of patients' records [2, 3]. Multiple techniques have been put forward for evaluating the integrity of case records [3-5]. Many audits and investigations have not turned up any evidence of deficiencies in medical record-keeping standards. The upkeep of medical records also has a lot of space for improvement [6].

The CRABEL score system, developed by CRAwford-BEresford-Lafferty [3], was one of the most beneficial for efficient documentation. The Annals of the Royal College of Surgeons of England described the CRABEL score as a way to rate each case note's quality about a set of objective criteria that were generated from the organization's published rule [3]. Evaluating case notes critically and providing feedback are crucial in raising their overall quality [4]. The evaluation of the operation notes' quality in comparison to this accepted norm identifies any issues and provides recommendations for improved operative notes [5]. 

A medical claim may be denied if records are not kept properly. Despite being aware of its importance, it is disheartening to observe that India is still in the early phases of record keeping. The adage "poor records mean poor defense, no records mean no defense" is wise to abide by [7]. With a greater emphasis now being placed on clinical results as well as patient happiness, quality and safety concerns have grown in significance in-hospital treatment. 

As a quality improvement (QI) strategy, health authorities and organizations prioritize audits by methodically assessing the care that is provided, identifying areas for improvement, and putting those improvements into practice [8]. Dhariwal et al. [9] implemented the CRABEL score to audit medical note-keeping at Morrison Hospital. All the clinicians in the maxillofacial unit received guidelines outlining the scoring methodology. Simplicity, dependability, and repeatability were the advantages of the CRABEL score. It was an effective and impartial way to increase the caliber of note-keeping and successful conduction of an audit. They suggested its implementation across all the maxillofacial units in the United Kingdom (UK) [9].

In our present study, we have screened and scored oral surgical case records of excisional biopsy cases of oral squamous cell carcinoma using the CRABEL scoring tool to verify and improve the documentation.

## Materials and methods

We conducted a case audit program based on the CRABEL score on our oral surgery department case records on Excision cases of oral squamous cell carcinoma operated at Saveetha Dental College and Hospitals, Chennai. Ethical clearance was obtained from the Institutional Human Ethical Committee with reference number IHEC/SDC/PhD/OPath-1954/19/TH-001. The audits were conducted from June 2023 - February 2024 for all Excisional biopsy cases utilizing the CRABEL Proforma [10].

**Figure 1:**
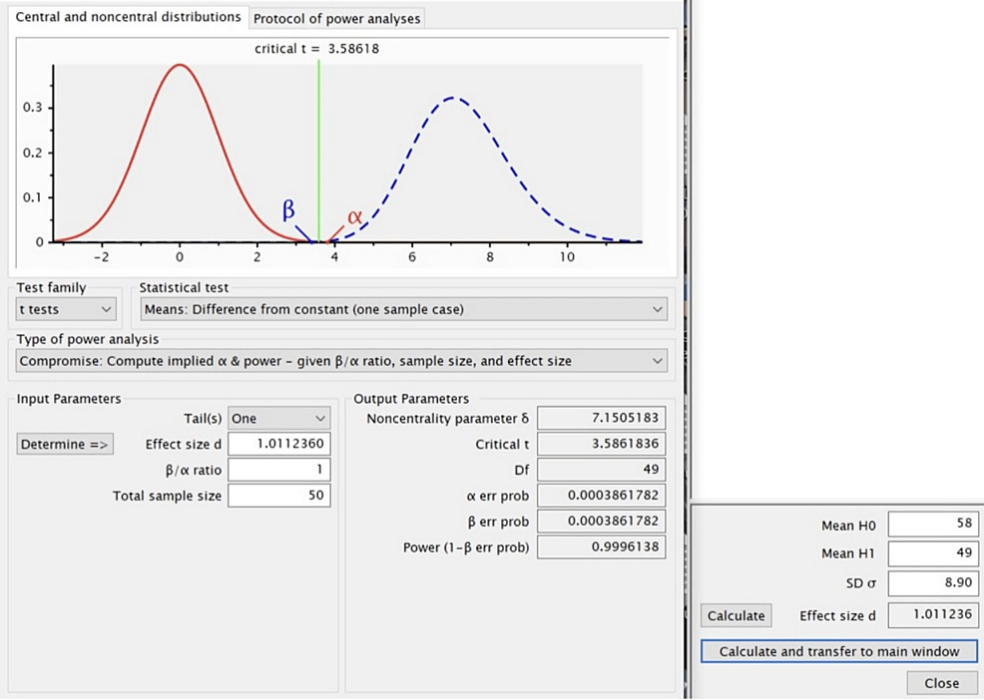
Sample size calculation using G*power software

The data were collected from Dental Information Archival Software (DIAS) of Saveetha Dental College and Hospitals, Chennai, after calculating the sample size from G*Power software (Version 3.1.9.7, Heinrich-Heine-Universität, Düsseldorf, Germany). The calculated sample size was 50 (Figure 1).

**Figure 2:**
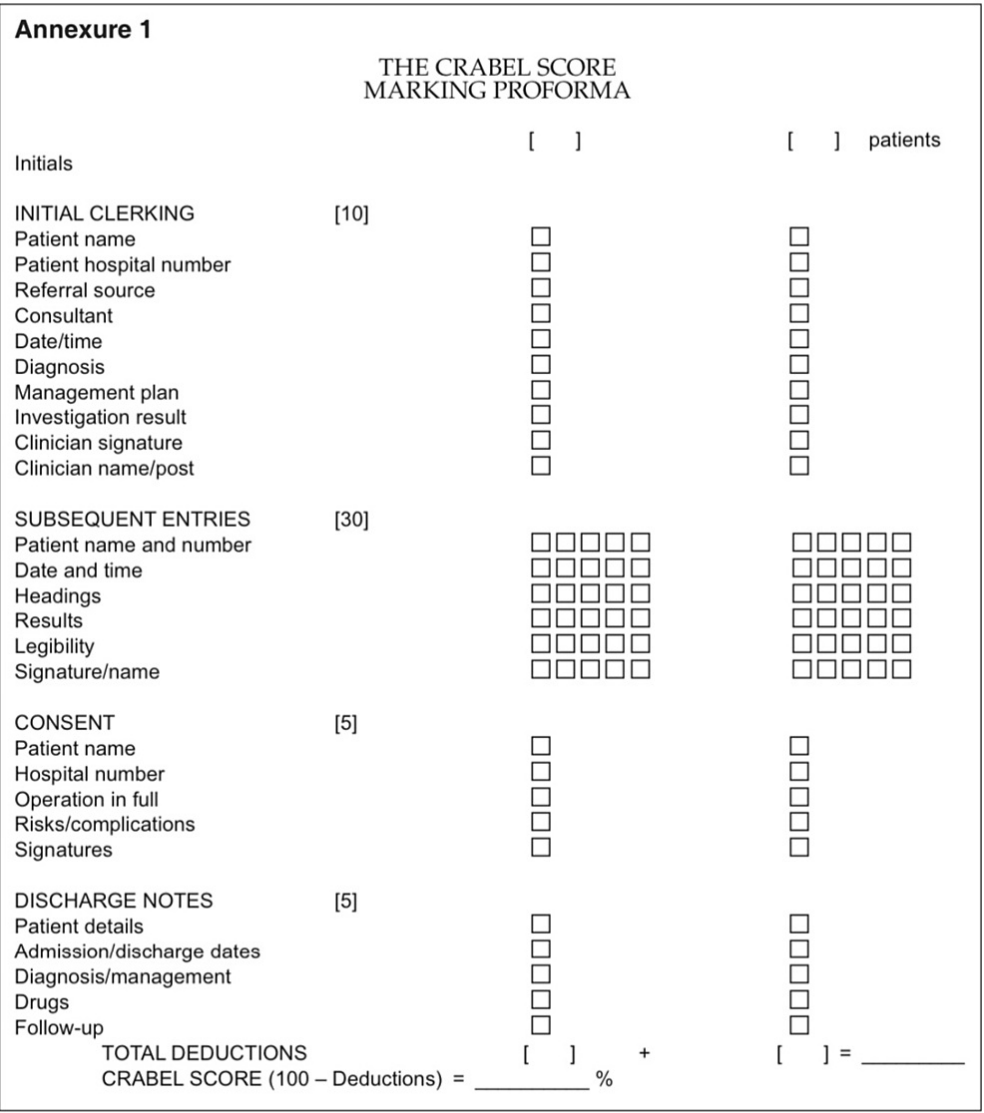
CRABEL score marking proforma The CRABEL score system developed by CRAwford-BEresford-Lafferty [3] and described by Crawford et al. [10]

The audit was performed based on the CRABEL criteria for all the excisional biopsy cases recorded during that period. The categories that have to be assessed are initial general examination, subsequent visits, updating schedule, post-operative instructions regarding post-operative recuperation, consent, discharge summary, and the documentation of guidance provided to the patient as described by Crawford et al. [10] (Figure 2).

Case notes of 52 consecutive excisional biopsies of oral squamous cell carcinoma cases were retrieved and analyzed. The activity was carried out using the CRABEL scoring sheet mentioned in Figure 2. Two oral pathologists were trained on CRABEL scoring and they individually assessed every case record and scored them. Each criterion was awarded a score as described by Crawford et al. [10].

Two consecutive case notes were evaluated. A total of 50 points were awarded to each case note. The questions were presented as yes/no choices. If the item was satisfied, one point was given; if not satisfied, none. Ten points were awarded for the first clerking section; 30 points for the subsequent entries, with up to six entries evaluated and five points awarded for each entry; five points were awarded for consent; and five points were awarded for the discharge letter. Every omission resulted in the loss of one point. To arrive at a final score, one point was subtracted from a beginning score of 100 for each omission (deficiency) [10-13]. If all the items were satisfied, then it was given a total score of 100. If any one item failed to satisfy, then it was reduced by one, and the total score became 99/100.

Based on the possible score and the score it received, the percentage was determined. For easy reference, correspondingly incomplete entries in the record were highlighted. The mean score was calculated for all of the records. The tabulated results were subjected to descriptive statistics with Statistical Package for Social Sciences (SPSS version 23.0, IBM Inc., Armonk, New York). Inter-observer agreement and reliability assessment were made using Kappa statistics. 

## Results

This study assessed 52 consecutive excisional biopsy case records of oral squamous cell carcinoma with CRABEL scoring. Two consecutive case records were scored for 50 points each. The initial clerking was 20 points in CRABEL criteria, and our audit results showed 18 points. The reference information was missing in the assessed records and was not found in any of the 52 records. Hence, the scoring for initial clerking was 18/20 in our audit. Consent was 10 points, subsequent entries were 30 points, and Discharge summary was 10 points in the CRABEL Criteria. Our audit results showed 10/10 points for consent, 30/30 points for subsequent entries, and 10/10 points for discharge summary. Thus, out of 100 points in the CRABEL criteria, our audit results showed 98/100.

**Figure 3:**
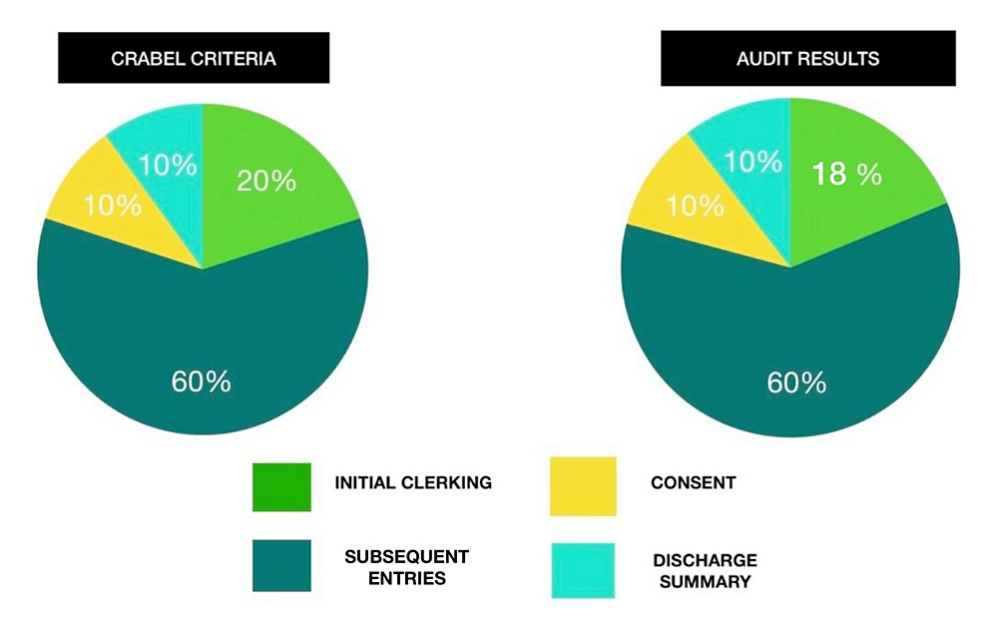
CRABEL score and our audit results Graphical representation of CRABEL criteria is shown on the left side and our audit results are shown on the right side.

There were no observed deficiencies in the consent (10%), subsequent entries (60%), and discharge summary (10%). There were deductions about initial clerking in our audit results (18%) as there was no evidence of a reference source versus (20%) in the original scoring. The diagrammatical representation of our case audit results is shown in Figure 3.

The audit's findings showed consistency among all oral surgery notes, with a score of 49 on the CRABEL scoring system assigned to each note and a total score of 98/100. We observed a score of 98/100 in all the assessed records (Table 1).

**Table 1:** CRABEL scores audit

CRABEL score audit	Total no of cases audited (n=52)
Initial score	18
Subsequent entries	60
Consent	10
Discharge summary	10
Total	98

It was noted to be the same between the two independent observers. Kappa statistics were performed for inter-observer agreement. The Kappa score attained was 1.0. There was no inter-observer bias in the scoring criteria and the mean values were similar to all the assessed records. 

## Discussion

Medical record-keeping has significantly improved with audits. One of the most important things in today's world of medical care is the aspect of consent. It is common knowledge that a patient must express informed permission before receiving medical care [11]. In the field of dentistry, specifically in excisional biopsy in oral surgery, standardized admission paperwork has been developed as a result of audit cycles, which have guaranteed that case-note quality is maintained as a top priority. The consent form should be filled with the patient's signature and ensure the patient's willingness to accept the treatment; this will help us avoid medicolegal cases. Discharge notes should be filled before the patient is released from the hospital [12, 13].

We observed a consistent score of 98/100 for all the 52 case records assessed. This consistent score showed that all of the reviewed cases had a high degree of documentation standards. There was only one change required in the initial clerking criteria, where the reference source was not mentioned clearly. Notably, deductions were made in the case note since the reference source's details were absent, which highlighted a particular area where the documentation needed to be enhanced. Raza conducted a CRABEL study on general medicine, general surgery, obstetrics, and gynecology, along with orthopedics, at a tertiary hospital in Gujarat, India. A case audit was carried out at General Surgery in Civil Hospital in Karachi, Pakistan, by Khan et al. [14]. They discovered that in 99%, 99%, and 89% of the notes, the names of the operating surgeon, the procedure's name, and post-operative instructions were mentioned, respectively. This high percentage was mainly because hospitals in Pakistan state employ a standard proforma template [14]. Likewise, we also found that 98% of the notes were according to standard proforma.

Root cause analysis (RCA) is a methodical process that focuses on identifying defects that could be improved for better performance and predictable results [15]. Case audits were performed to identify and rectify the defects in making good high-quality oral surgery case records. Every patient must be given utmost attention at every review, and the quality of life (QOL) must be considered. Following surgery, there is a marked decline in quality of life (QOL), which necessitates ongoing monitoring with appropriate motivation and careful dietary monitoring by the professional nutritionist. To enhance the patient's health-related quality of life, the surgeon should therefore strive for a minimal resection followed by the best repair required for the patient's age and illness stage. Sufficient counseling must be provided [16]. This study states that the QOL of the patient should be considered before we decide on the treatment plan, and in this audit, we came to know that they are maintaining regular follow-ups for the patient, and counseling for the patient is also provided.

The essential idea, which is founded on autonomy, was first expressed in the 1947 Nuremberg Code. The German Nazi regime's experiments and medical atrocities led to the adoption of the Nuremberg Code shortly after World War II [17]. Obtaining the voluntary and informed consent of human subjects is mandated under the code. Similar to this, the World Medical Association's 1964 Declaration of Helsinki highlights the significance of gaining freely given informed consent for medical research by fully disclosing to subjects the study's objectives, procedures, expected benefits, potential risks, and associated discomforts [18]. The quality of the consent form was excellent. This is partly due to the department's strong emphasis on getting informed permission, the close collaboration of specialist staff, and the routine evaluation of consent paperwork.

Our investigation into the condition of surgical notes at our hospital was prompted by the growing emphasis on management duties and audits. Financial and medical retrospective audits will not be accepted until hospital patient files are properly maintained. To address the issue, a large infusion of resources is needed. Hospitals that have not yet conducted in-depth analyses of the information in their notes urgently need to do so [19]. Missing referral information impacts the quality of care. It hinders the understanding of patient history. Complete records with referral information improve diagnosis and treatment. To prevent losses in terms of financial compensation and for medicolegal grounds, pertinent facts about the surgery, any follow-up treatments, and any problems should also be meticulously noted. It is important to educate resident trainees on their significance and to pursue their inclusion in the surgical curriculum [20]. In this audit, we observed that the Oral surgery department was functioning to its fullest to maintain its standards in all the criteria and maintain its integrity in patient care.

The quality of surgical notes has significant economic and medicolegal effects in addition to its intrinsic medical implications. Additionally, properly documented records can be helpful for research advancement and auditing, which can enhance patient care delivery [21]. We have observed that the referral from where the patient came from was left out of the documentation. In hospitals, the referral source plays a critical role in facilitating proper care and guaranteeing a smooth treatment continuum. Referrals to sophisticated intervention-capable hospitals enable the handling of complicated cases. To improve treatment outcomes and service quality, referral sources are essential in matching patients with the appropriate resources. Furthermore, recommendations promote cooperation among medical professionals, guaranteeing patients receive thorough and interdisciplinary care. Hospitals could access a network of specialist services through referral sources that may not be available locally, raising patient satisfaction and the standard of treatment overall.

Including the referral source in initial clerking also helps healthcare providers identify potential gaps in care or areas where additional follow-up may be necessary. For example, if a patient has been referred by a specialist for a specific condition, the healthcare provider can ensure that the patient is receiving appropriate ongoing care and follow-up appointments with the specialist. Furthermore, referral sources can provide valuable feedback and insights into the patient's care, which can help healthcare providers improve their services and overall patient satisfaction.

Other methodologies like e-CRABEL and The Surgical Tool for Auditing Records (STAR) are modified versions of the CRABEL system that are more tailored to surgical records auditing and have been used in other studies with promising improvements [21, 22]. Surgical Hospital Audit of Record Keeping (SHARK) is a new audit and teaching tool for junior doctors based on the Royal College of Surgeons guidelines to anonymously score the different surgical teams' medical records within a hospital [23]. Pediatricians have proposed to improve the quality of documentation by structuring their notes using subjective, objective, assessment, and planning (SOAP) format [24].

We have employed only CRABEL scoring for our case audit. We have also compared only excisional cases of oral squamous cell carcinoma in this study. We are already pursuing a comparative analysis of different audit methods across various oral surgical records including trauma, orthognathic surgeries, cleft surgeries, and cancer surgery. A comparative study between other methods can give us a better understanding of improving the quality of case records.

## Conclusions

Our study shows that CRABEL scoring can be used to audit oral surgery case notes of excisional biopsy for oral squamous cell carcinoma. The referral source is a crucial piece of information that should be included in initial clerking to ensure effective communication, coordination, and continuity of care for patients. The key to streamlined patient care is improving the surgical record the quality with all the relevant patient details, which will foster better patient outcomes and ultimately enhance the quality of life.
